# Monitoring Antioxidant Preservation in Microwave-Dried Tea Using H_2_O_2_-Responsive Electrochemical Sensor

**DOI:** 10.3390/foods15030595

**Published:** 2026-02-06

**Authors:** Jiaoling Wang, Hao Li, Xinxin Wu, Xindong Wang, Xinai Zhang

**Affiliations:** 1 Key Laboratory of Modern Agricultural Equipment, Nanjing Institute of Agricultural Mechanization, Ministry of Agriculture and Rural Development, Nanjing 210014, China; 2 School of Food and Biological Engineering, Jiangsu University, Zhenjiang 212013, China; 19351703383@163.com (H.L.); 19825549348@163.com (X.W.); w1341501173@163.com (X.W.)

**Keywords:** electrochemical sensor, hydrogen peroxide, catechin, antioxidant activity, tea extract, microwave-assisted drying

## Abstract

Considering the demand for nutritional assessment and product quality control in the tea industry, this work develops an effective electrochemical sensor based on gold nanoparticles electrodeposited onto a zeolitic imidazolate framework (Au/MOF(Zn)) for evaluating the antioxidant activity of tea subjected to microwave-assisted drying (MAD) through hydrogen peroxide (H_2_O_2_) scavenging. The MOF(Zn) enables uniform deposition of AuNPs, which significantly enhances the electrocatalytic oxidation of H_2_O_2_. The fabricated sensor exhibits a wide linear detection range from 400 μM to 1.8 mM for H_2_O_2_ with a correlation coefficient of 0.9983. The experimental results demonstrate acceptable selectivity, with signal interference <5% from common tea compounds like inorganic ions, sugars, and organic acids. Electrochemical methods, including cyclic voltammetry (CV) and differential pulse voltammetry (DPV) analysis, were employed to quantify H_2_O_2_ by measuring oxidation currents in phosphate-buffered saline (PBS, pH 7.0). The relative standard deviation (RSD) for repeatability and reproducibility was 5.1% and 6.8%, respectively, confirming high reliability. This sensor was successfully applied to assess antioxidant capacity in tea extracts obtained from fresh leaves subjected to microwave-assisted drying under varying power and duration. Results indicate that increasing microwave power enhances antioxidant activity, while prolonged drying at low power initially increases activity (peaking at 120 s) but reduces it upon extended exposure. Optimal antioxidant preservation was achieved at 120 s. This real-time, reliable sensing strategy offers theoretical foundations for optimizing tea processing parameters to preserve bioactive compounds, particularly polyphenols like catechins, thereby improving tea quality and health benefits.

## 1. Introduction

Tea, one of the most widely consumed beverages worldwide [1,2,3,4], has transcended cultural and geographical boundaries to become a staple in daily diets, cherished not only for its sensory appeal but also for its diverse health-promoting properties. Central to its biological activity are bioactive compounds, particularly polyphenols such as catechins, emerging as the primary contributors to its functional benefits [5,6,7]. These polyphenols collectively account for 65–80% of the total polyphenol content in tea, with their concentration varying by tea type (e.g., green, black, oolong) and processing methods. In this respect, polyphenols stand out for their exceptional antioxidant potency, exhibiting high capacity to scavenge reactive oxygen species (ROS), including hydrogen peroxide (H_2_O_2_), superoxide anions, and hydroxyl radicals [8,9,10]. By neutralizing these oxidative molecules, tea polyphenols mitigate oxidative stress, a pathological hallmark linked to the development and progression of chronic diseases [1,11,12,13]. The nutritional and commercial value of tea products is thus inherently tied to the stability and bioavailability of these antioxidants, making the accurate evaluation of antioxidant capacity a critical step in both academic research and industrial quality control. Recent studies have emphasized that factors such as cultivation conditions, harvesting time, and post-harvest handling can significantly alter polyphenol profiles [14,15,16,17], underscoring the need for standardized analytical protocols to ensure consistent product quality.

Against this backdrop, tea processing techniques [18,19,20], especially drying methods, emerge as pivotal determinants of phytochemical preservation and bioactivity retention [21,22]. Traditional drying methods [23,24,25], such as sun drying and hot-air drying, have been employed for centuries but often suffer from limitations: sun drying is weather-dependent and prone to contamination, while prolonged hot-air drying may cause thermal degradation of heat-sensitive compounds like catechins. In contrast, microwave-assisted drying (MAD) has gained traction in recent decades due to its unique advantages, including rapid and uniform heating, reduced processing time, and improved energy efficiency compared to conventional methods [26,27,28]. The mechanism of MAD involves generating heat through molecular friction [29,30,31], which accelerates moisture evaporation and minimizes processing duration, theoretically reducing exposure to destructive high temperatures. However, the intense thermal energy and potential localized overheating in MAD systems raise concerns about the integrity of bioactive components, as polyphenols are known to degrade under excessive heat or prolonged processing [32,33]. To address this paradox, researchers have begun exploring variables such as microwave power, drying time, and initial moisture content to optimize MAD conditions [34,35]. Nevertheless, the lack of real-time monitoring tools has hindered precise control over these parameters, resulting in inconsistent outcomes across studies. Consequently, the development of reliable, sensitive, and real-time analytical patterns is essential to revolutionize tea processing that balances efficiency with maximal bioactive retention, ensuring the delivery of high-quality products with enhanced health benefits to global consumers.

Conventional methods for evaluating antioxidant activity include spectrophotometric assays such as 2,2-diphenyl-1-picrylhydrazyl (DPPH), 2,2′-azino-bis(3-ethylbenzothiazoline-6-sulfonic acid) (ABTS), and ferric reducing antioxidant power (FRAP) [36,37,38,39,40]. These techniques are widely applied due to their simplicity and accessibility, relying on colorimetric changes to estimate radical scavenging or reducing capacity [41]. However, despite their popularity, these approaches often require extensive sample pretreatment, prolonged reaction times, and the use of unstable or hazardous reagents, which limit their suitability for rapid, real-time, or on-site analysis. Moreover, they provide only an indirect assessment of antioxidant activity based on bulk reactions, lacking mechanistic insight into electron transfer kinetics. A significant drawback is their low specificity; interfering compounds such as sugars, organic acids, or pigments in complex matrices like tea extracts can distort results, leading to overestimation or false positives. Additionally, these assays are generally endpoint-based, making them unable to capture dynamic changes in antioxidant levels during processing. These limitations underscore the urgent need for more efficient, selective, and quantitative analytical strategies that offer real-time monitoring capability and higher accuracy in evaluating antioxidant behavior in food and beverage systems.

Electrochemical sensing offers a highly promising alternative for antioxidant evaluation [42,43,44], combining high sensitivity, rapid response, low cost, and operational simplicity [45,46]. Unlike conventional spectrophotometric patterns, electrochemical techniques directly monitor electron transfer processes in redox reactions [47,48,49,50], enabling real-time, quantitative analysis of reactive species such as H_2_O_2_. In recent years, nanozymes, nanomaterials with enzyme-mimicking catalytic properties [51,52], have emerged as powerful tools in electrochemical biosensing due to their stability, tunable activity, and large surface-to-volume ratios. Particularly, metal–organic frameworks (MOFs) exhibit intrinsic peroxidase-like and catalase-like activities [53,54,55,56], making them ideal nanozymes for H_2_O_2_ assay. Encouragingly, our previous studies have successfully demonstrated that nanozyme-based electrochemical sensors can rapidly and accurately detect H_2_O_2_ in complex matrices. This foundational work provides a robust platform for extending such sensing strategies to dynamic systems, such as monitoring oxidative stress changes during food processing. These advancements highlight the great potential of integrating nanozymes with electrochemistry for developing reliable, sensitive, and real-time analytical methods in food research.

In this study, we developed an electrochemical sensing platform based on gold nanoparticle electrodeposited onto a zeolitic imidazolate framework (Au/MOF(Zn)/GCE) for assessing the antioxidant activity of dried tea via H_2_O_2_ analysis. The MOF(Zn) provides a porous, high-surface-area support that facilitates dense and uniform deposition of AuNPs, significantly enhancing the electrocatalytic oxidation of H_2_O_2_. By measuring the resulting current signal in a phosphate-buffered solution, the sensor enables precise quantification of H_2_O_2_ content over a wide linear range with high selectivity and reproducibility. This capability was strategically applied to evaluate the antioxidant activity of tea extracts obtained from fresh leaves subjected to microwave-assisted drying under varying power and duration conditions. Specifically, the scavenging of H_2_O_2_ by tea polyphenols, particularly catechins, leads to a measurable decrease in the electrochemical signal, which correlates directly with antioxidant capacity. Thus, by comparing the suppression of H_2_O_2_ current before and after tea extract addition, we can dynamically assess how different drying parameters influence bioactive compound preservation. This approach offers a rapid, reliable, and insightful method for optimizing tea processing to maximize health-promoting properties.

## 2. Materials and Methods

### 2.1. Materials and Reagents

Zinc nitrate hexahydrate (Zn(NO_3_)_2_·6H_2_O) and 2-methylimidazole were obtained from Aladdin Reagent Co., Ltd. (Shanghai, China). Methanol, chloroauric acid (HAuCl_4_), sodium sulfate (Na_2_SO_4_), sulfuric acid (H_2_SO_4_), hydrogen peroxide (H_2_O_2_), phosphate-buffered saline (PBS, pH 7.0), potassium hexacyanoferrate(II) (K_4_[Fe(CN)_6_]), potassium ferricyanide (K_3_[Fe(CN)_6_]), anhydrous ethanol and citric acid were obtained from Sinopharm Chemical Reagent Co., Ltd. (Shanghai, China). Catechin standard and sucrose were purchased from Macklin Biochemical Co., Ltd. (Shanghai, China). All chemicals were used as received without further purification, and ultrapure water (18.2 MΩ·cm) was prepared using a Milli-Q system (Millipore, St. Louis, MI, USA).

### 2.2. Apparatus

Morphological characterization of the samples was conducted using a JSM-7800F field emission scanning electron microscope (SEM, Hitachi High-Technologies Corporation, Hitachinaka, Japan). Samples were sputter-coated with a 5 nm thin layer of gold (E-1045 ion sputter, Hitachi, Hitachinaka, Japan) to enhance conductivity. All electrochemical experiments were performed on a CHI 660D electrochemical workstation (Chenhua Instrument Co., Ltd., Shanghai, China) at room temperature. A conventional three-electrode system was employed, consisting of a platinum wire as the auxiliary, a saturated calomel electrode (SCE) as the reference, and a glassy carbon electrode (GCE, Φ = 3 mm) as the working electrode. Before use, GCE was polished sequentially with 0.3 and 0.05 μm alumina slurry on microcloth pads, followed by ultrasonic cleaning in anhydrous ethanol and ultrapure water for 5 min each. Cyclic voltammetry (CV) was performed at a scan rate of 50 mV s^−1^, and differential pulse voltammetry (DPV) measurement was performed with an amplitude of 50 mV, pulse width of 0.05 s.

### 2.3. Preparation of MOF(Zn) Nanostructure

A zinc-based metal–organic framework was synthesized via a solvothermal method with minor modifications [57,58,59]. Briefly, Zn(NO_3_)_2_·6H_2_O (1.49 g, 5 mmol) and 2-methylimidazole (0.82 g, 10 mmol) were separately dissolved in 50 mL of anhydrous methanol under magnetic stirring. The 2-methylimidazole solution was then slowly added dropwise to the Zn(NO_3_)_2_ solution, and the mixture was stirred continuously at room temperature for 24 h to ensure complete coordination. The resultant MOF(Zn) precipitate was collected via differential centrifugation and washed sequentially with methanol to remove unreacted ligands and metal precursors. Each washing step involved resuspending the precipitate in fresh methanol, sonicating for 5 min, and centrifuging again. After the final wash, the product was transferred to a vacuum drying oven and dried at 50 °C for 12 h to remove residual solvent. The resulting MOF(Zn) was a white crystalline powder, which was stored in a desiccator at room temperature until further use.

### 2.4. Preparation of Au/MOF(Zn)-Decorated Sensing Interface

The MOF(Zn) powder was dispersed in distilled water to prepare a homogeneous 2 mg/mL suspension via ultrasonic treatment. Following the pretreatment procedure for the glassy carbon electrode (GCE) [60,61], 10 μL of the MOF(Zn) suspension was drop-cast onto the pretreated GCE surface using a micropipette (Eppendorf, Hamburg, Germany), ensuring uniform coverage. The electrode was dried in a vacuum oven at 50 °C for 2 h to immobilize MOF(Zn), yielding the MOF(Zn)/GCE intermediate.

The MOF(Zn)/GCE was immersed in an electrodeposition bath containing 1 mM HAuCl_4_, 0.01 M Na_2_SO_4_ (supporting electrolyte), and 0.01 M H_2_SO_4_. A CHI 660D electrochemical workstation was used for gold nanoparticles (AuNPs) electrodeposition at a constant potential of −0.2 V vs. SCE, deposition time of 800 s. After deposition, the electrode was rinsed with ultrapure water to remove loosely adsorbed AuNPs and dried under nitrogen. The resulting modified electrode was denoted as Au/MOF(Zn)/GCE. This method is optimized for tea extracts processed via microwave drying and may require recalibration for other matrices or antioxidant mechanisms.

### 2.5. Electrochemical Sensing Toward H_2_O_2_

The electrochemical properties of the modified electrodes (bare GCE, MOF(Zn)/GCE, Au/GCE, and Au/MOF(Zn)/GCE) were first characterized via CV measurement in a redox probe solution containing 5 mM [Fe(CN)_6_]^4−^/^3−^ (1:1 molar ratio) and 0.1 M KCl as the supporting electrolyte. CV scans were performed over a potential range of −0.8 to 0.8 V at a scan rate of 50 mV s^−1^. The peak-to-peak separation (ΔE_p_) and redox peak currents (I_p_) were recorded to assess electron transfer kinetics at the electrode/electrolyte interface. The catalytic activity toward H_2_O_2_ reduction was evaluated via CV in 0.1 M PBS buffer (pH 7.0) with 5 mM H_2_O_2_. The reduction peak current of H_2_O_2_ was measured, and the background current (in PBS without H_2_O_2_) was subtracted to obtain the net response. Under optimal conditions, differential pulse voltammetry (DPV) was employed to quantify H_2_O_2_ concentration. H_2_O_2_ standard solutions were prepared in 0.1 M PBS (pH 7.0), and DPV scans were recorded with the following parameters: potential range of −0.6 to 0.2 V, amplitude of 50 mV, pulse width of 0.05 s, pulse period of 0.5 s, and quiet time of 2 s.

### 2.6. Electrochemical Evaluation of Antioxidant Activity of Tea

Fresh tea leaves were harvested from a local garden (Zhenjiang, China) and processed immediately using a domestic microwave oven (Midea MM720CGF-PW, 800 W maximum power; Midea, Foshan, China). Drying was performed under four different power levels (200, 400, 600, 800 W) for 60 s, and at different drying times (30–210 s) was also studied at 200 W. After rinsing with ultrapure water and blotting dry, 0.5 g portions of fresh leaves were weighed into porcelain crucibles. Drying was performed using a domestic microwave oven under different power levels for the same duration, or alternatively, applying the same power level for varying drying times. After drying, tea polyphenols were extracted via ultrasonic-assisted extraction. Briefly, add 6 mL of anhydrous ethanol to the dried tea powder and perform ultrasonic extraction for 30 min in an ultrasonic bath, followed by filtration and storage protected from light for further analysis. The extracted tea products are then added in H_2_O_2_ solution, and electrochemical signals are acquired using the developed sensing strategy.

## 3. Results and Discussion

### 3.1. Principle of Electrochemical Sensing Toward Antioxidant Activity of Tea

**Scheme 1:**
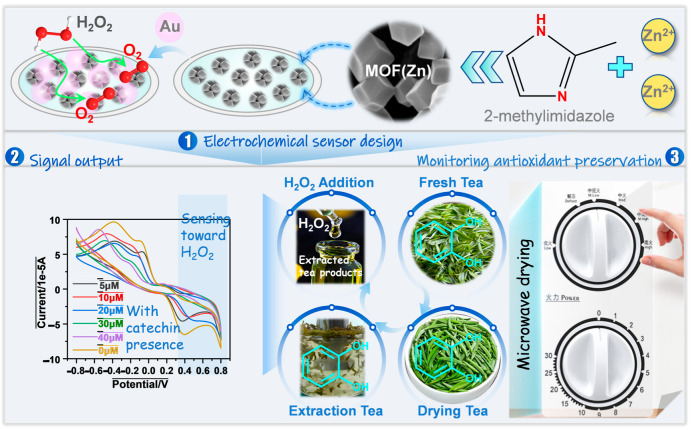
Schematic illustration of designing the sensing interface and monitoring the antioxidant activity of tea under microwave drying via electrochemical H_2_O_2_ assay.

A sensitive electrochemical sensor was developed for evaluating the antioxidant activity of tea based on H_2_O_2_ scavenging (Scheme 1). MOF(Zn) was synthesized via a solvothermal method and then drop-cast onto the electrode surface, followed by electrodeposition of AuNPs to form the Au/MOF(Zn)-modified GCE. The Au/MOF(Zn) composite exhibited enhanced electrocatalytic activity toward H_2_O_2_ oxidation due to the high conductivity of AuNPs and the large surface area of MOF(Zn). A linear relationship between oxidation current and H_2_O_2_ concentration was established, enabling a quantitative assay. Catechin, a major tea polyphenol, was used to assess antioxidant capacity by measuring the decrease in H_2_O_2_ current response. This electrochemical strategy was applied to evaluate the antioxidant activity of tea extracts obtained from fresh leaves dried under varying microwave powers and durations. Antioxidant activity increased with higher microwave power, while prolonged drying at low power initially enhanced, then reduced activity. The Au/MOF(Zn)-based sensor enables rapid, reliable evaluation of tea antioxidant capacity, offering theoretical foundations for optimizing microwave-assisted drying parameters to preserve bioactive components in tea processing. While the sensor provides a robust measure of H_2_O_2_ scavenging capacity (a dominant antioxidant pathway in tea), it does not capture the full spectrum of antioxidant activity (e.g., superoxide dismutation or lipid peroxidation inhibition).

### 3.2. Characterization of MOF(Zn) Properties

**Figure 1:**
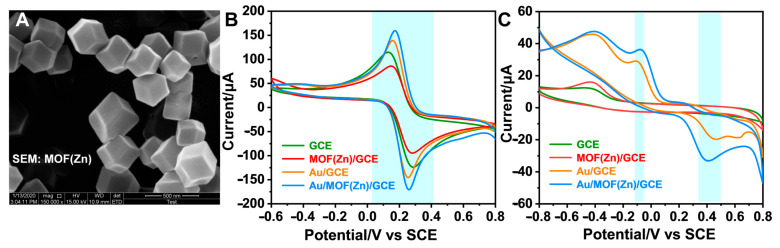
(**A**) SEM image of zeolitic imidazolate framework (MOF(Zn)). (**B**) CV curves (vs. SCE) from different modified electrodes in [Fe(CN)_6_]^4−/3−^. (**C**) CV curves (vs. SCE) from different modified electrodes in 0.1 M PBS containing 5 mM H_2_O_2_.

The morphology of the as-synthesized MOF(Zn) was first analyzed via SEM. As shown in Figure 1A, MOF(Zn) exhibits a uniform rhombic dodecahedral crystal structure with sharp edges and smooth surfaces. The particle size distribution is relatively narrow, ranging from 200 to 500 nm in diameter, which aligns with the solvent-mediated synthesis protocol using methanol as the solvent and 2-methylimidazole as the ligand. SEM images also confirm the absence of significant aggregation, indicating that the ultrasonic dispersion step effectively stabilized the MOF(Zn) particles.

The current responses under four different conditions were measured by CV measurements in a 5 mM [Fe(CN)_6_]^4−/3−^ solution containing 0.1 M KCl. As shown in Figure 1B, the bare electrode (green curve) exhibits a pair of redox peaks around 0.3 V, indicating that ferricyanide undergoes redox reactions on the bare electrode. When the electrode is modified with MOF(Zn) (red curve), the peak current decreases, which may be due to the low conductivity of MOF(Zn) or MOF(Zn) hindering the contact between the electrode and [Fe(CN)_6_]^4−/3−^ in the solution, thereby weakening the current response. After modification with AuNPs (orange curve), the peak current increases, possibly attributed to the high conductivity of AuNPs or the promotion of the interaction between the electrode and [Fe(CN)_6_]^4−/3−^ in the solution, thus enhancing the current response. However, after the GCE is modified with the MOF(Zn) and AuNPs composite (blue curve), the peak current of the redox peaks is significantly enhanced, surpassing that of the single-modified electrode. These results indicate the successful construction of a more effective Au/MOF(Zn)/GCE sensing interface.

The CV curves of bare GCE, MOF(Zn)/GCE, and Au/MOF(Zn)/GCE in 0.1 M PBS buffer solution containing 5 mM H_2_O_2_ were compared to gain insight into the electrochemical detection performance of each electrode for H_2_O_2_. Figure 1C shows that in a 0.1 M PBS buffer solution, after the addition of H_2_O_2_, bare GCE (green curve) and MOF(Zn)/GCE (red curve) did not produce redox peaks, indicating that H_2_O_2_ does not undergo obvious redox reactions on GCE and MOF(Zn)/GCE. Au/GCE (orange curve) generated a pair of redox peaks around −0.2 V and 0.3 V. For the GCE modified with Au/MOF(Zn) (blue curve), the electrical signals were significantly enhanced at approximately −0.2 V and 0.4 V. These results indicate that AuNPs have electrocatalytic activity for H_2_O_2_, and the large specific surface area of MOF(Zn) can allow more gold nanoparticles to attach to the electrode surface, enhancing the current signal.

### 3.3. Optimization of Experimental Parameter

**Figure 2:**
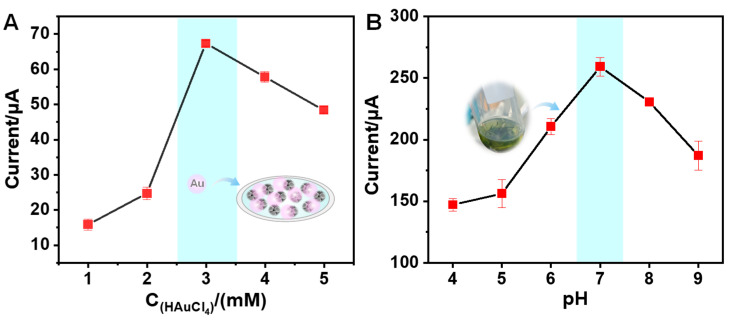
Effects of (**A**) HAuCl_4_ content and (**B**) pH value on current response of electrochemical sensor toward H_2_O_2_ assay.

To maximize the sensitivity and stability of the Au/MOF(Zn)/GCE sensor for H_2_O_2_ detection, key experimental parameters were systematically optimized, including the concentration of HAuCl_4_ during electrodeposition and pH of the supporting electrolyte. The concentration of HAuCl_4_ directly influences the size, density, and distribution of electrodeposited AuNPs, which are critical for catalytic activity. HAuCl_4_ concentrations of 1, 2, 3, 4, and 5 mM were tested. As shown in Figure 2A, the current response increased with HAuCl_4_ concentration from 1 to 3 mM, reaching a maximum at 3 mM, likely due to enhanced AuNP loading and surface coverage. At concentrations above 3 mM, the response decreased, possibly due to AuNP aggregation and reduced active surface area. Thus, 3 mM HAuCl_4_ was selected as optimal.

The pH of the supporting electrolyte is a critical parameter influencing the structural stability of nanomaterials, the catalytic activity of the sensing interface, and the chemical stability of H_2_O_2_ itself. To determine the optimal pH, the electrochemical response of the Au/MOF(Zn)/GCE sensor toward H_2_O_2_ was evaluated in 0.1 M PBS buffers with pH values ranging from 4.0 to 9.0. As shown in Figure 2B, the current response increased gradually from pH 4.0 to 7.0, reaching a maximum at pH 7.0, and then decreased when pH was above 7.0. Although H_2_O_2_ is most chemically stable under weakly acidic conditions (pH 3.5–4.5), alkaline environments (pH > 7.0) accelerate its decomposition into O_2_ and H_2_O, reducing the effective concentration available for detection. Thus, PBS buffer with pH 7.0 was selected as the optimal condition, balancing both H_2_O_2_ stability and maximum electrochemical signal response.

### 3.4. Electrochemical Sensing Toward H_2_O_2_

**Figure 3:**
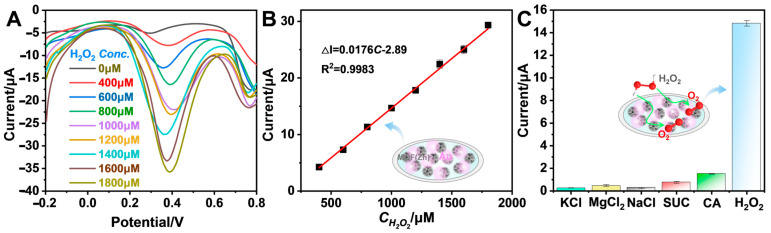
(**A**) DPV current response from the proposed sensor toward H_2_O_2_ at different concentrations. (**B**) The corresponding standard curve between the current response and H_2_O_2_ concentration. (**C**) Specificity testing of 1 mM H_2_O_2_ against KCl, MgCl_2_, NaCl, sucrose (SUC), and citric acid (CA).

Under optimal experimental conditions, electrochemical analysis was performed to detect a standard H_2_O_2_ solution at different concentrations. As shown in Figure 3A, the DPV peak current increased with the increasing H_2_O_2_ concentration. Within the detection range of 400 μM–1.8 mM, there was a good linear relationship between the increased current response (ΔI) and H_2_O_2_ concentration (Figure 3B). The linear fitting equation was ΔI = 0.0176C − 2.89 (C is H_2_O_2_ concentration) and the correlation coefficient reached 0.9983. Additionally, the repeatability (intra-assay precision) of the proposed sensor was assessed by applying one modified electrode for a 1 mM H_2_O_2_ assay. The current response for five consecutive trials is 14.6 μA, 16.2 μA, 15.7 μA, 16.8 μA, and 15.9 μA, and the relative standard deviation (RSD) is calculated to be 5.1%, indicating acceptable repeatability.

The reproducibility (inter-assay precision) of the Au/MOF(Zn)/GCE sensor was evaluated by fabricating five independent modified electrodes under identical conditions and measuring their DPV responses toward 1 mM H_2_O_2_ in 0.1 M PBS (pH 7.0). The relative standard deviation (RSD) of the peak currents was calculated to be 6.8% (*n* = 5), indicating satisfactory fabrication reproducibility. This uniformity is attributed to the controlled electrodeposition of AuNPs and the stable immobilization of MOF(Zn) on the GCE surface. To assess the anti-interference ability of the sensor, the DPV responses toward 1 mM H_2_O_2_ were measured in the presence of common interfering substances, including inorganic ions (KCl, MgCl_2_, NaCl, 10 mM each) and organic molecules (sucrose (SUC), citric acid (CA), 1 mM each), which are typically present in tea extracts or biological samples. As shown in Figure 3C, the current response to H_2_O_2_ (defined as 100%) was significantly higher than the responses to the interfering substances (<5% of the H_2_O_2_ signal). This indicates that the Au/MOF(Zn)/GCE sensor exhibits acceptable specificity for H_2_O_2_, likely due to the selective catalytic activity of AuNPs toward H_2_O_2_ reduction and the molecular sieving effect of the MOF(Zn) framework, which limits the diffusion of larger interfering molecules.

### 3.5. Electrochemical Study on Antioxidant Activity

**Figure 4:**
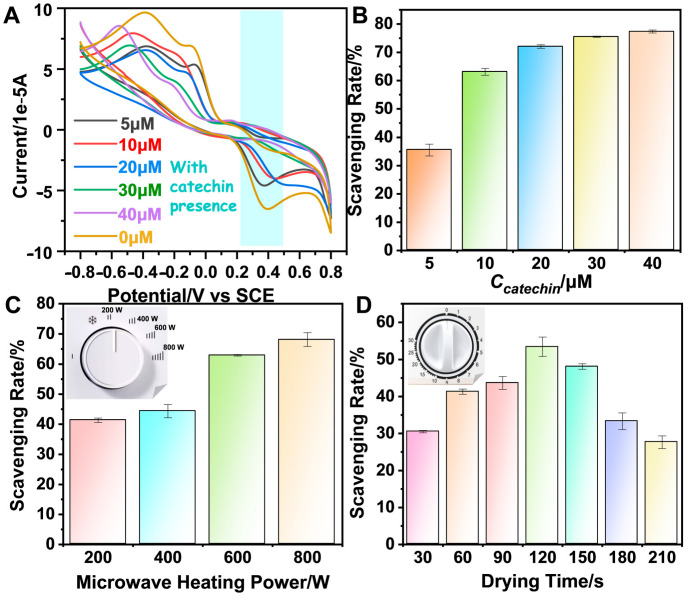
(**A**) CV curves (vs. SCE) for 1.8 mM H_2_O_2_ assay with the presence of catechin at different concentrations. (**B**) The corresponding scavenging effect of catechin against H_2_O_2_. Scavenging effect of tea leaves under different microwave heating powers for 60 s (**C**) and that under Low-temperature heating for different durations (**D**).

Tea polyphenols, particularly catechins (which constitute 65–80% of total tea polyphenols, are well recognized for their potent antioxidant activity via ROS scavenging. To validate the utility of the Au/MOF(Zn)/GCE sensor for quantifying antioxidant capacity, catechin, a representative tea polyphenol with high ROS-scavenging efficiency, was used as a model antioxidant. Herein, catechin solutions (0, 5, 10, 20, 30, and 40 μM) were prepared in 0.1 M PBS (pH 7.0) and mixed with an equal volume of 3.6 mM H_2_O_2_ (final H_2_O_2_ concentration 1.8 mM). The mixtures were incubated at 25 °C for 10 min to allow catechin-H_2_O_2_ reaction, then analyzed via DPV using the Au/MOF(Zn)/GCE sensor. As shown in Figure 4A, the current response around 0.4 V (vs. SCE) for the H_2_O_2_ assay decreased with the presence of catechin at an increasing concentration, confirming that catechin effectively scavenges H_2_O_2_. Correspondingly, the scavenging efficiency (%) of catechins on H_2_O_2_ was analyzed by calculating the scavenging rates at different catechin concentrations, using the following formula:

$$Scavenging\;\;rate=\frac{I_{P}\left(control\right)-I_{P}\left(exp\right)}{I_{P}\left(control\right)}$$

in which *Ip* (control) is the current response for the 1.8 mM H_2_O_2_ assay without catechin, *Ip* (*exp*) is the current response for the 1.8 mM H_2_O_2_ assay with the presence of catechin at different concentrations. As seen from Figure 4B and Table 1, the sensor can effectively evaluate catechin-mediated H_2_O_2_ scavenging, providing a direct readout of antioxidant activity.

To investigate the antioxidant capacity of tea samples during microwave drying, fresh leaves were first harvested for testing. A 0.5 g sample was weighed and placed in a microwave oven for drying at low, medium, high, and overhigh power levels, each for 60 s. After drying, 6 mL of anhydrous ethanol was added to the tea samples, followed by ultrasonic extraction for 30 min. The resulting sample was then filtered and stored in the dark for later use. During the experiment, the proposed sensor was first used to scan a DPV curve in a solution containing 1.8 mM H_2_O_2_. Subsequently, 1 mL of the tea sample was added to the tested H_2_O_2_ solution, and another DPV measurement was performed to calculate the scavenging rate of the tea sample. Figure 4C and Table 2 illustrate the differences in scavenging rates after drying for 60 s at different microwave heating powers, showing that antioxidant activity increased with higher drying power.

As for evaluating the drying duration for fresh tea leaves, weigh 0.5 g of a fresh tea sample and place it in a microwave oven for drying at low power for 30 s, 60 s, 90 s, 120 s, 150 s, 180 s, and 210 s, respectively. Subsequently, add 6 mL of anhydrous ethanol, perform ultrasonic extraction for 30 min, filter the solution, and store it in the dark for later use. Aspirate 1 mL of the pretreated sample, add it to 4 mL of H_2_O_2_ solution, incubate for 60 s, and then proceed with detection. Use a blank control group without the tea sample addition, and finally calculate the scavenging rate. As depicted in Figure 4D and Table 3, under low-power conditions, the antioxidant activity in tea leaves initially increased and then decreased with prolonged drying time, reaching optimal activity at 120 s of drying.

## 4. Conclusions

This work successfully developed an effective electrochemical sensor based on AuNPs electrodeposited onto a MOF(Zn) for evaluating the antioxidant activity of microwave-dried tea via H_2_O_2_ scavenging. The rhombic dodecahedral structure of MOF(Zn) provided a stable platform for AuNP immobilization, with the resulting Au/MOF(Zn) nanocomposite significantly enhancing the electrocatalytic reduction in H_2_O_2_ through synergistic effects: MOF(Zn) offered high-surface-area and molecular sieving properties, while AuNPs facilitated rapid electron transfer and catalytic activity. The sensor exhibited good analytical performance, including a wide linear detection range, high selectivity with minimal interference from common tea compounds, and excellent repeatability and reproducibility. These characteristics make it a reliable tool for real-time and accurate quantification of H_2_O_2_ in tea extracts. When applied to evaluate the antioxidant activity of tea subjected to different microwave-assisted drying conditions, the sensor revealed valuable insights. It was found that increasing microwave power generally enhances antioxidant activity, while the effect of drying duration at low power is more complex, initially increasing and then decreasing activity. The optimal drying time of 120 s was identified for preserving the maximum antioxidant capacity. This research not only provides a new and efficient analytical method for assessing antioxidant activity in tea but also offers practical guidance for optimizing microwave-assisted drying parameters in the tea industry. By enabling real-time monitoring of antioxidant levels during processing, this sensor has the potential to improve the quality and health benefits of tea products, ensuring that consumers receive tea with enhanced bioactive compound content. Future work could focus on further expanding the application of this sensor to other food systems and exploring its long-term stability under different storage and operational conditions.

## Figures and Tables

**Table 1:** H_2_O_2_ scavenging rate under different catechin concentrations.

Catechin Conc. /μM	Scavenging Rate/%			Average/%
	Three-Measured Values			
5	33.32	35.71	37.51	35.51
10	61.91	64.29	63.17	63.12
20	72.69	71.48	72.11	72.09
30	75.42	75.36	75.82	75.53
40	77.21	77.97	76.93	77.37

**Table 2:** Scavenging effect of tea leaves under different microwave heating modes for 60 s.

Microwave Heating Power/W	Scavenging Rate/%			Average/%
	Three-Measured Values			
200	42.03	41.45	40.54	41.31
400	46.21	44.9	41.94	44.35
600	63.11	63.02	62.61	62.91
800	65.82	70.38	68.17	68.12

**Table 3:** Scavenging effect of tea leaves under low-temperature heating for different durations.

Drying Time/s	Scavenging Rate/%			Average/%
	Three-Measured Values			
30	30.77	30.1	30.58	30.48
60	41.94	41.45	40.54	41.31
90	44.4	41.68	44.9	43.66
120	50.41	54.65	55.22	53.43
150	47.45	47.96	48.87	48.09
180	30.8	35.24	33.82	33.29
210	29.5	26.2	27.18	27.63

## Data Availability

The original contributions presented in this study are included in the article. Further inquiries can be directed to the corresponding authors.

## Abbreviations

Microwave-assisted drying: MAD; cyclic voltammetry: CV; differential pulse voltammetry: DPV; hydrogen peroxide: H_2_O_2_; gold nanoparticles: AuNPs; zeolitic imidazolate framework: MOF(Zn); phosphate-buffered saline: PBS; relative standard deviation: RSD; reactive oxygen species: ROS; 2,2-diphenyl-1-picrylhydrazyl: DPPH; 2,2′-azino-bis(3-ethylbenzothiazoline-6-sulfonic acid: ABTS; ferric reducing antioxidant power: FRAP; metal–organic frameworks: MOFs; glassy carbon electrode: GCE; Zinc nitrate hexahydrate: Zn(NO_3_)_2_·6H_2_O; hloroauric acid: HAuCl_4_; sodium sulfate: Na_2_SO_4_; sulfuric acid: H_2_SO_4_; potassium hexacyanoferrate(II): K_4_[Fe(CN)6]; potassium ferricyanide: K_3_[Fe(CN)_6_]; scanning electron microscope: SEM.
